# Tenogenesis of Decellularized Porcine Achilles Tendon Matrix Reseeded with Human Tenocytes in the Nude Mice Xenograft Model

**DOI:** 10.3390/ijms19072059

**Published:** 2018-07-15

**Authors:** Anke Lohan, Benjamin Kohl, Carola Meier, Gundula Schulze-Tanzil

**Affiliations:** 1 Charité-Universitätsmedizin Berlin, Corporate Member of Freie Universität Berlin, Humboldt-Universität zu Berlin, and Berlin Institute of Health, Department of Traumatology and Reconstructive Surgery, Campus Benjamin Franklin, Hindenburgdamm 30, 12203 Berlin, Germany; anke.lohan@charite.de (A.L.); benjamin.kohl@charite.de (B.K.); carola.meier@charite.de (C.M.); 2 Institute of Anatomy, Paracelsus Medical University, Salzburg and Nuremberg, Prof.-Ernst-Nathan Strasse 1, 90419 Nuremberg, Germany

**Keywords:** extracellular tendon matrix, polyglycolic acid, decellularization, Achilles tendon, human tenocytes, tenogenesis

## Abstract

Cultivation of autologous human tenocytes in a cell-free xenogenic extracellular tendon matrix (xECM) could present an approach for tendon reconstruction. The aim of this study was to achieve tendon-like tissue formation by implanting decellularized porcine Achilles tendons recellularized with human hamstring tendon-derived tenocytes into nude mice. The structure of decellularized xECM was histologically monitored before being dynamically reseeded with human tenocytes. After 6–12 weeks in vivo, construct quality was monitored using macroscopical and histological scoring systems, vitality assay and quantitative DNA and glycosaminoglycan (GAG) assays. For comparison to tendon xECM, a synthetic polyglycolic acid (PGA) polymer was implanted in a similar manner. Despite decellularized xECM lost some GAGs and structure, it could be recellularized in vitro with human tenocytes, but the cell distribution remained inhomogeneous, with accumulations at the margins of the constructs. In vivo, the xECM constructs revealed in contrast to the PGA no altered size, no inflammation and encapsulation and a more homogeneous cell distribution. xECM reseeded with tenocytes showed superior histological quality than cell-free implanted constructs and contained surviving human cells. Their DNA content after six and 12 weeks in vivo resembled that of native tendon and xECM recellularized in vitro. Results suggest that reseeded decellularized xECM formed a tendon-like tissue in vivo.

## 1. Introduction

Tendon healing is a time-consuming process, often leading to a biomechanically-inferior repaired tissue. In some cases of tendon and ligament defects, surgical reconstruction is demanded to reestablish functionality due to substantial tissue loss. So far, developed synthetic biomaterials tested for tendon substitution cannot fully restore tendon functionality and withstand repetitive tension. Hence, autologous tendon grafts have to be harvested in most cases from other sites of the patient, associated with donor site morbidities [1,2,3,4,5]. Therefore, the use of a natural and functional cell-free tendon extracellular matrix (ECM) could present a suitable approach for tendon defect reconstruction. The removal of cells from allogenic or xenogenic tendons is necessary to abolish immunogenic motifs to avoid allogenic rejection after implantation. One crucial advantage of using a natural tendon ECM for repair is the typical alignment of the collagen type I fiber bundles in this ECM and its anisotropy. Both factors have been shown to support the tenogenic differentiation of precursor cells [6,7,8]. This unique property of natural tendon ECM scaffolds could allow the recruitment of stem cells such as mesenchymal stromal cells (MSCs) for tendon repair. Decellularized porcine-derived ECMs have been established for many other applications, and some of them have already entered the clinic. Approaches include porcine urinary bladder (urinary bladder matrix, aCell), porcine heart valves (Synergraft®, FDA approved) [9], porcine small intestinal mucosa (SIS), porcine superflexor tendon [10,11], porcine dermis for abdominal and pelvic hernia reconstruction [12] and porcine cornea [13]. SIS is used in preclinical trials for Achilles (AS) tendon [14], anterior cruciate ligament [15] and rotator cuff repair [16]. Porcine tissues are easily available and share similarities with human tissues [17].

Several approaches have been developed to decellularize various tendon or ligament tissues [10,11,18,19,20,21] and to reseed the cell-free ECM in vitro [22]. The main problems are an inhomogeneous cell distribution in response to reseeding due to the high density of the ECM, which limits nutrition and cell penetration, and furthermore, dependent on the decellularization protocol, growth factor and glycosaminoglycan (GAG) deprivation in decellularized tendons and ligaments [23,24,25]. However, so far, published in vivo studies documenting the performance of diverse xECM are mostly restricted to the use of MSCs, or even cell-free xECMs [11,26,27], but only a few adopt human tenocytes for recellularization [21,28,29,30,31]. MSCs could undergo osteogenesis when implanted into mouse, rat or rabbit tendons [32].

Therefore, the present study in the nude mice model was undertaken to directly compare the in vitro and in vivo outcome of a decellularized porcine AS tendon and for comparison, a synthetic biomaterial (polyglycolic acid (PGA)) seeded or not with differentiated human tenocytes. PGA has been selected here as a control since it revealed overall biocompatibility in a formerly-performed AS tendon defect model in rabbit [33]. 

## 2. Results

### 2.1. Decellularization of the Porcine AS Tendon xECM

**Figure 1:**
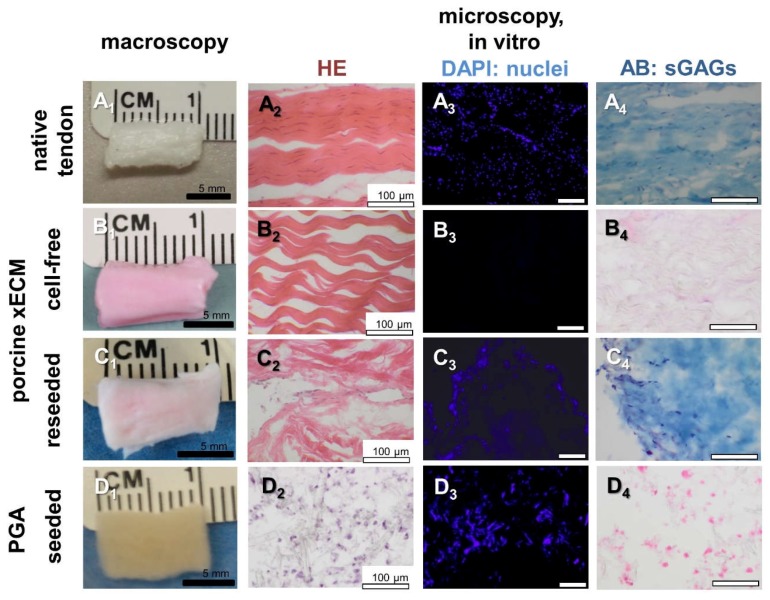
Macroscopical and histological appearance of native porcine AS tendon and decellularized xECM or PGA seeded or not with human tenocytes after one week in vitro. (**A_1_**–**D_1_**) Macroscopical images of native AS tendon, cell-free and reseeded (one week) xECM (**A_1_**–**C_1_**) and seeded (one week) PGA (**D_1_**). (**A_2_**–**D_2_**) HE staining. (**A_3_**–**D_3_**) DAPI: cell nuclei are blue. (**A_4_**–**D_4_**) AB staining (sGAGs: sulfated glycosaminoglycans are blue). Representative images are shown. Scale bars 5 mm (A_1_–D_1_) and 100 µm (**A_2_**–**D_4_**).

After decellularization, reseeding and one week in vitro culturing, no major size and shape changes occurred in xECM and PGA (Figure 1A_1_–D_1_). Compared to the cell content in native tissue, decellularization of the xECM led to complete cell disappearance, as shown by HE, DAPI and AB stainings. No remaining cell nuclei could be detected by the stainings (Figure 1B_2–4_). However, some structure loss such as clefts and loose bundles of collagen fibers were detected by the histological stainings (Figure 1B_2_,B_4_). A loss of GAGs compared to the normal content in the native AS tendon was demonstrated in this study by the AB staining (Figure 1A_4_,B_4_).

### 2.2. Recellularization of the Porcine AS Tendon xECM with Human Tenocytes In Vitro

Human tenocytes were able to adhere to the decellularized xECM (Figure 1C_2_–C_4_). However, cell distribution remained inhomogeneous with accumulation of the cells at the ends of the constructs. Recellularization led to some recovery of the GAG content in the xECM, as shown by AB staining (Figure 1C_4_). In the xECM, rounded and ovoid cell nuclei could be detected, whereas in PGA constructs, round cell nuclei dominated. 

### 2.3. Macroscopical Construct Quality after Implantation into the Nude Mice

**Figure 2:**
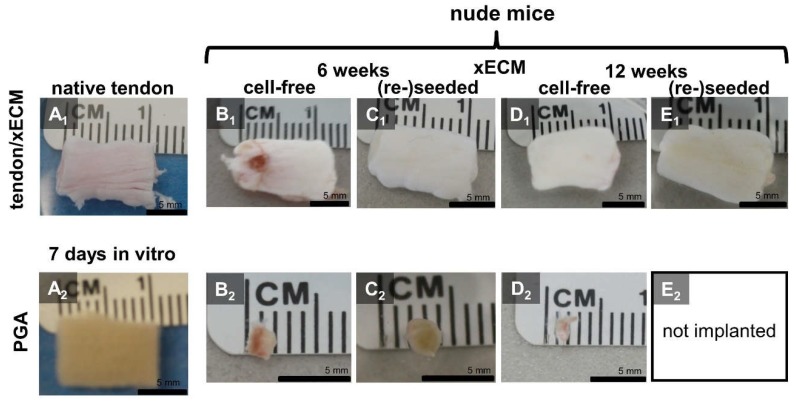
Macroscopical appearance of native porcine AS tendon and decellularized xECM or PGA seeded or not with human tenocytes after six and 12 weeks in vivo. (**A_1_**) native tendon, (**A_2_**) PGA after seven days in vitro. (**B_1_**,**D_1_**) cell-free xECM, (**C_1_**,**E_1_**) recellularized xECM. (**B_1,2_**–**C_1,2_**) Explanted from nude mice after six weeks. (**D_1,2_**–**E_1,2_**) Explanted from nude mice after 12 weeks.

**Figure 3:**
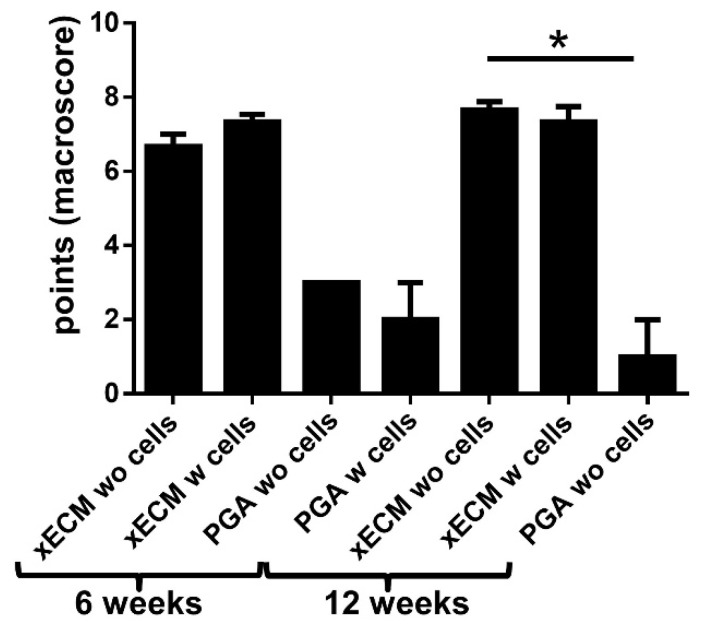
Results of macroscopical scoring of decellularized xECM or PGA seeded or not with human tenocytes after six and 12 weeks in vivo. PGA: polyglycolic acid. w: with, wo: without cells. * *p* < 0.05.

Compared with the native tendon pieces used for decellularization, reseeding and culturing procedures of the xECM constructs, irrespective of whether with or without cells, caused no major alterations of size after six and 12 weeks in vivo implantation (Figure 2A_1_–E_2_). All explanted xECM constructs had a white and glossy appearance with a smooth surface. The surface was smoother than in the xECM before in vivo implantation. In contrast, PGA constructs became rapidly smaller, irrespective of whether reseeded or not; hence, for 12 weeks, only cell-free PGA was implanted to spare animals (Figure 2A_2_–E_2_). The macroscopical scoring results of cell-free PGA after 12 weeks in vivo were significantly inferior compared to the cell-free xECM at the same time (Figure 3). The differences between seeded and unseeded PGA and xECM were not significant.

### 2.4. Histological Construct Quality after Implantation into the Nude Mice

**Figure 4:**
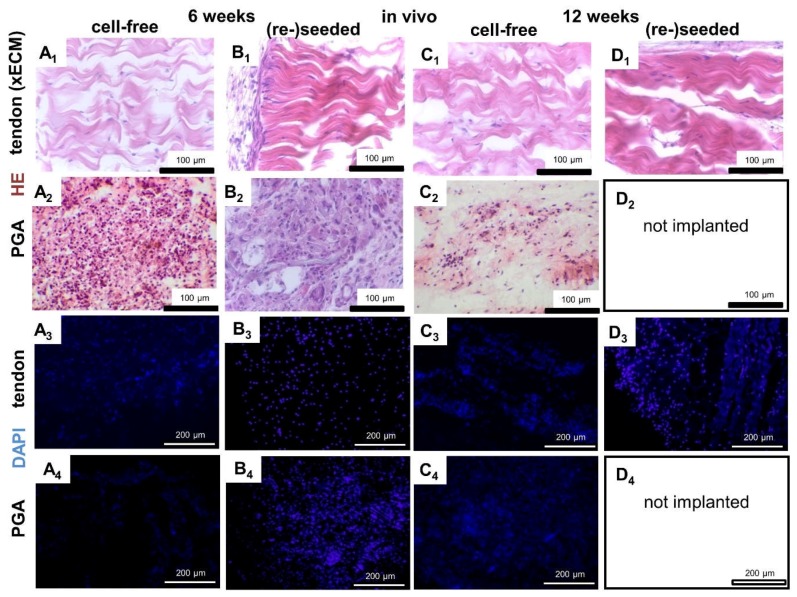
Histological appearance of decellularized xECM seeded or not with human tenocytes and seeded PGA and implanted for six or 12 weeks in nude mice. Histological staining of xECM (**A_1_**–**D_1_**,**A_3_**–**D_3_**) and PGA (**A_2_**–**D_2_**,**A_4_**–**D_4_**) after in vivo implantation for six weeks (**A_1_**–**B_4_**) and 12 weeks (**C_1_**–**D_4_**). (**A_1_**–**D_2_**) HE staining. (**A_3_**–**D_4_**) DAPI staining. xECM or PGA were implanted either cell-free (**A_1–4_**,**C_1–4_**) or reseeded with human tenocytes (**B_1–4_**,**D_1–4_**). Scale bars 100 µm (**A_1_**–**D_2_**) and 200 µm (**A_3_**–**D_4_**).

**Figure 5:**
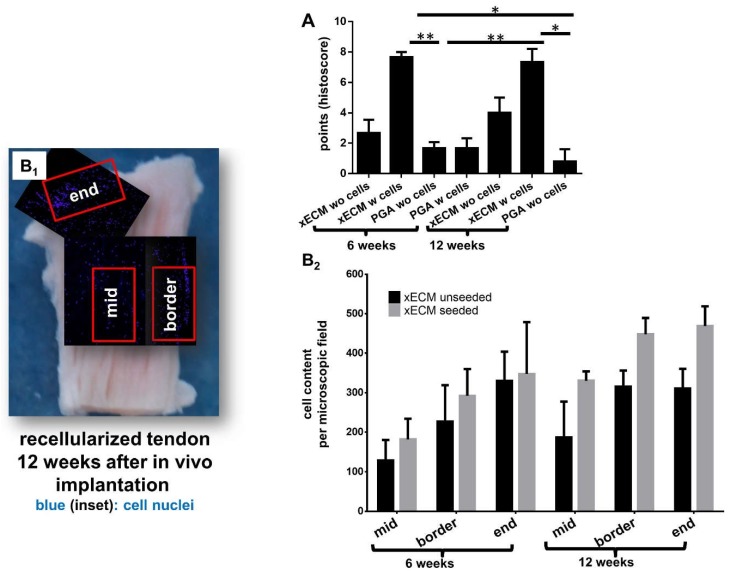
Results of histopathological scoring and cell numbers of decellularized xECM or PGA seeded or not with human tenocytes six and 12 weeks after in vivo incubation. (**A**) Results of histological scoring, (**B_1_**) areas included in cell number calculations and (**B_2_**) counted cells based on DAPI staining. DAPI-stained cell nuclei were counted in an equal-sized tissue area of a cryo-section. *n* = 3. PGA: polyglycolic acid. wo: without cells, w: with cells. * *p* < 0.05, ** *p* < 0.01.

Since the PGA constructs explanted from the mice were too small, only histology could be performed. Only a thin membrane surrounded the tendon xECM constructs (Figure 4B_1_). No inflammatory or foreign body response or major encapsulation could be detected histologically in the xECM after in vivo implantation (Figure 4). PGA constructs revealed a thicker capsule. However, the PGA constructs became smaller, and later (12 weeks), some of them were completely resorbed. An inhomogeneous cell distribution could be observed in vitro. Most of the cells had rather round or ovoid cell nuclei in PGA and xECM compared to native tendon, which contained more elongated cell nuclei. PGA constructs contained also inflammatory cells, particularly when cell-free implanted for six weeks (Figure 4). However, after in vivo implantation (Figure 4 and Figure 5B_1_), the cell distribution was much more homogeneous compared to samples cultured only in vitro (Figure 1). 

xECM constructs seeded with human tenocytes showed superior histological score values at six and 12 weeks compared with the non-seeded xECM (not significant) and all PGA constructs (significant) (Figure 5A). Constructs seeded with cells before implantation had on average also a higher cell content (not significant) than non-seeded constructs, irrespective of the area monitored (mid, border or end of the construct; Figure 5B_2_). Compared with the cell content in the mid of both types of xECM constructs, higher numbers of cells could be detected at the borders and ends (Figure 5B_1_).

**Figure 6:**
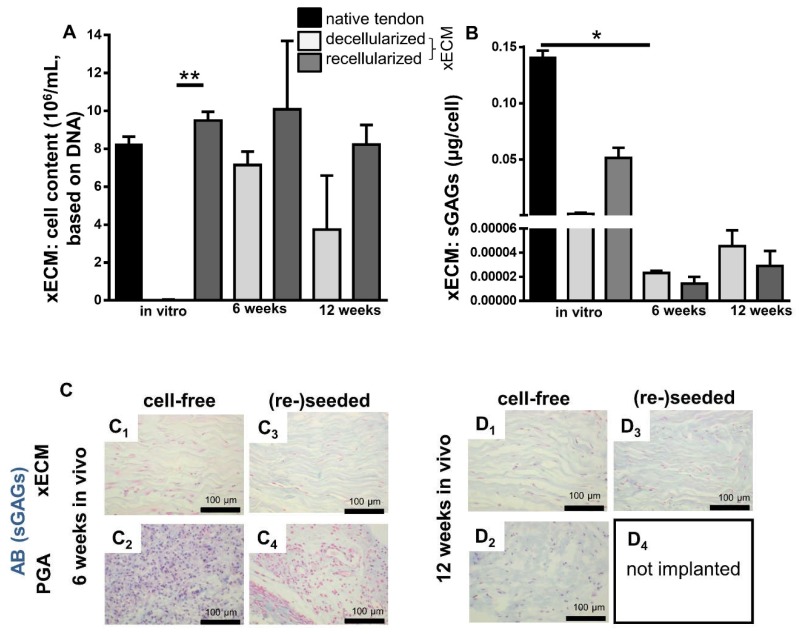
Cell and GAG content in native, decellularized and recellularized xECM in vitro and in vivo six and 12 weeks after implantation in the nude mice. (**A**) Cell content. Cell numbers were calculated by the assumption of 7.7 ng DNA per cell. (**B**) sGAG content assessed using the DMMB assay. (**C**) AB staining after six weeks (**A_1_**–**B_2_**) and 12 weeks (**C_1_**–**D_2_**) in vivo. Cell-free implanted samples (**A_1–2_**,**C_1–2_**) and samples, recellularized before implantation (**B_1–2_**,**D_1–2_**). Scale bars 100 μm. sGAGs: sulfated glycosaminoglycans., * *p* < 0.05, ** *p* < 0.01.

The DNA content after six and 12 weeks in vivo was not significantly different from that observed after recellularization for only seven days in vitro (Figure 6A) and did also not differ from that observed in native tendon. However, the constructs seeded with tenocytes before implantation revealed a higher cell content calculated from DNA at the time of explantation than the non-reseeded constructs (Figure 6A, not significant). Some minor fatty and connective tissue infiltration was detectable in xECM. Interestingly, the development of rows of univacuolar adipocytes from multivacuolar precursors was detectable (Supplemental Figure S1). In comparison to this, the native tendon also naturally contained a few groups of adipocytes.

### 2.5. Comparison of GAG Content In Vitro and In Vivo

The GAG content was monitored quantitatively using the DMMB assay. It was only examined in the xECM since the explanted PGA samples became too small to perform this analysis. The GAG content in recellularized xECM in vitro was higher compared with the cell-free xECM, but it was still lower compared with native tendon (Figure 6B). Interestingly, the GAG content of recellularized samples was significantly lower after six weeks of in vivo incubation compared with the in vitro cultured recellularized samples and native tendons. It showed a slight increase after 12 weeks in vivo compared to six weeks. However, no major difference existed between cell-free and reseeded constructs in vivo at the six- and 12-week observation time points (Figure 6B).

Nevertheless, AB staining supported the results from the DMMB assay. It revealed a faint GAG staining in recellularized and decellularized xECM samples after 12 weeks in vivo (Figure 6D_1_,D_3_), which was higher compared to six weeks (Figure 6C_1_,C_3_), but lower than after in vitro cultivation (Figure 1A_4_,C_4_).

### 2.7. Comparison of αSMA in Native Tendon and in Decellularized or Recellularized xECM Implanted in Nude Mice for Six and 12 Weeks

**Figure 7:**
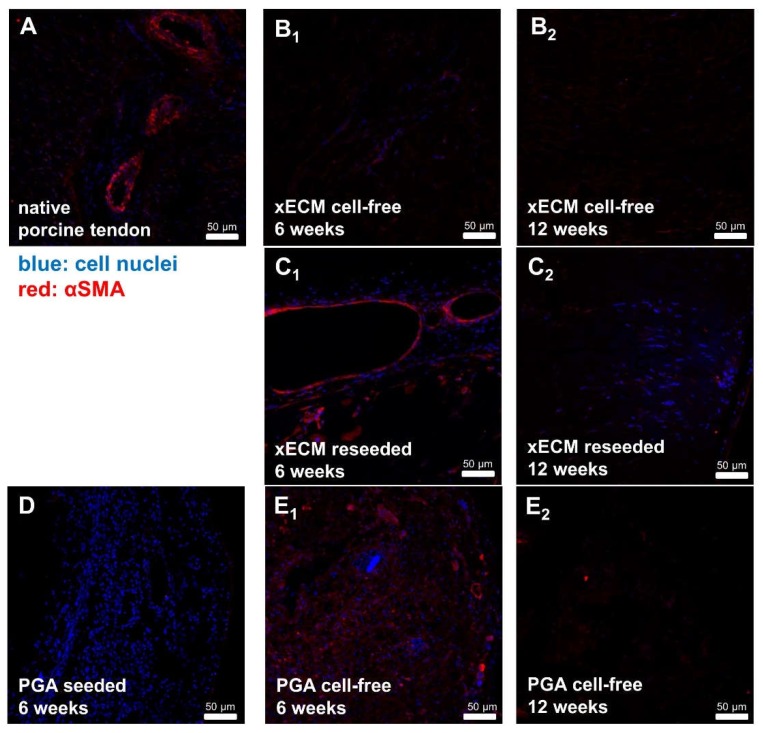
αSMA expression in native and recellularized xECM in vivo six and 12 weeks after implantation in the nude mice. (**A**) Native porcine AS tendon, (**B**,**C**) xECM decellularized and implanted in nude mice for six (**B_1_**) and 12 weeks (**B_2_**) or xECM recellularized with human tenocytes and implanted in nude mice for six (**C_1_**) and 12 weeks (**C_2_**), immunolabeled for αSMA using a Cy3-coupled secondary antibody. (**D**) PGA, recellularized and implanted for six weeks. (**E**) PGA, implanted cell-free for six (**E_1_**) and 12 (**E_2_**) weeks (w) in vivo.

αSMA containing blood vessels could be detected in native tendons (epitenon region) and in the capsule region of recellularized and cell-free implanted xECM and PGA samples explanted from the nude mice after six and 12 weeks. This observation indicated that blood vessel accessed the implants (Figure 7). 

### 2.8. Detection of Human Cell Nuclei in Recellularized xECM and Implanted in Nude Mice for Six and 12 Weeks

**Figure 8:**
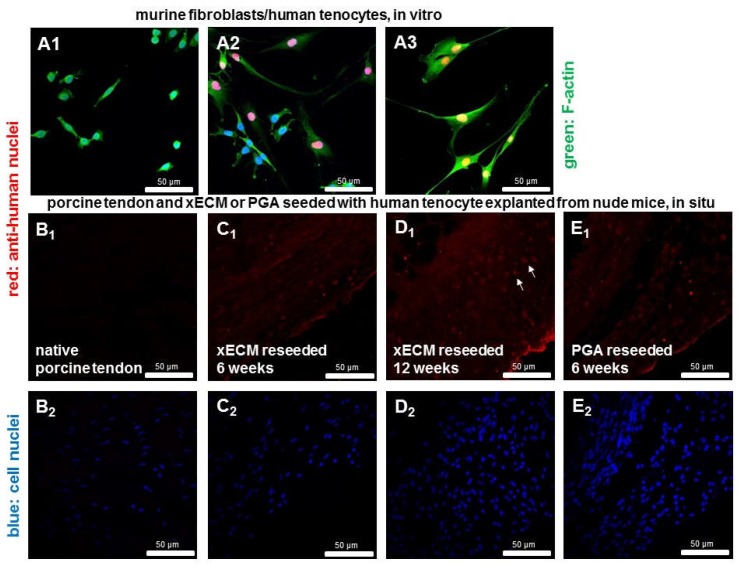
Detection of human cell nuclei recellularized xECM in vivo six and 12 weeks after implantation in the nude mice. (**A_1–3_**) Human hamstring tenocytes and the murine subcutaneous fibroblast cell line (L929) were immunolabeled for human cell nuclei (48 h of culture). (**A_1_**) L929, (**A_2_**) co-culture consisting of L929 and human tendon fibroblasts and (**A_3_**) human tendon fibroblasts. (**A_1_–A_3_**) Green, Phalloidin-Alexa488 staining of F-actin cytoskeleton. (**A_1–_E_1_**) Anti-human cell nuclei immunolabeling (red) to mark human-derived cell nuclei. (**A_1–3_**,**B_2_**–**E_2_**) DAPI staining to show all cell nuclei. (**B_1–2_**) Native porcine AS tendon, (**C**,**D**) xECM decellularized and recellularized with human tenocytes and implanted in nude mice for six (**C**) and 12 weeks (**D**), immunolabeled for anti-human nuclei and Cy3-coupled secondary antibody. D_1_: arrows mark cell nuclei (red) of human cells. (**E_1–2_**) PGA recellularized for six weeks.

The anti-human cell nuclei staining allowed distinguishing between human tenocytes and mouse-derived subcutaneous fibroblasts (Figure 8A_1–3_). It showed remaining human tenocytes in the constructs implanted for six or 12 weeks in the murine host (Figure 8B_1_–E_2_).

## 3. Discussion

The xECM was characterized by a high stability of size in contrast to the PGA after six and 12 weeks in vivo, irrespective of whether seeded with cells or not. Minor shape changes arose from pressure, shear forces and shift movement in the subcutaneous compartment in the mice. Accordingly, at the histological level, the tendon’s overall structure was maintained including the wavy pattern of longitudinally-running collagen fiber bundles, except for the formation of some clefts between the ECM bundles. As reported in a previous study with decellularized tendon xECM [22] some loss of structure can be observed in decellularized xECMs, probably as a result of biochemical changes induced by components of the decellularization buffers, but possibly also mechanically due to shear forces arising during the dynamical culturing procedure in a tilting tube roller. It has been shown that the main collagen architecture is conserved in ligament tissue decellularized with a comparable protocol [19]. A volume loss of various cell-rich tissues could be observed previously due to cell loss [34]. However, since tendon contains only few cells (5–10% of the tissue volume), no major volume loss could be detected in the present study. The biomechanical properties of the decellularized xECM are dependent on the protocol and are in most cases not severely compromised [23,25,35,36]; hence, they were not investigated here. Nevertheless, decent tissue clefts are of some advantage to favor cell penetration and distribution during and after reseeding [24]. It is also known that natural ECMs lose some of their GAGs during decellularization [20,23]. Collagen fibers are covered by GAGs in the ECM; hence, the loss of GAG might impair the coherence of collagen fiber bundles contributing to cleft formation. On the other hand, it is known that GAG-containing proteoglycans inhibit cell adhesion [37], suggesting that GAG loss could present some advantage for reseeding the xECM. The data show that some GAG recovery can be achieved by cell colonization of the xECM. Interestingly, a low GAG content could be detected after in vivo implantation of the constructs. This might be explained by an interaction of mice-derived host cells with the xECM as an attempt to remodel it. Mice host cells release several proteases such as matrix metalloproteinases capable of degrading the xECM. However, in contrast to the PGA, the xECM resists this attack, maintaining its size. PGA has been shown to be degraded if implanted without colonizing cells or in cases when the colonizing cells are unable to produce ECM rapidly enough covering the PGA fibers [38]. PGA revealed some inflammatory cells. In contrast, only a few inflammatory cells and foreign body cells could be found in the xECM; therefore, no major inflammatory response could be detected underlining the biocompatibility of the xECM. Only a very thin connective tissue capsule surrounded the xECM constructs at the time of explantation responsible for the shinier macroscopical appearance compared with the xECM before implantation. This connective tissue layer resembled histologically an epitenon. It remained thin and consisted of connective tissue with fibroblasts and some adipocytes. One could also suspect that it might result from some foreign body response evoked by the xECM. However, inflammatory cells were mostly lacking, and foreign body giant cells could not be detected in the capsule. Compared to the xECM, the capsule surrounding the explanted PGA was substantially thicker. 

Cell survival could be proven in the xECM before implantation, and vital cells were also detected after explantation. A much more homogeneous cell distribution was observed in the xECM after in vivo incubation compared with in vitro culture. The cell content in the recellularized xECM as calculated from the DNA content revealed no major differences when compared with the native tissue, neither in vitro, nor after in vivo implantation. In addition, also the cell-free constructs revealed thorough cell colonization, suggesting the immigration of host-derived extrinsic fibroblasts from the subcutaneous mouse tissue. Therefore, a tendon-like tissue could be attained by the xECM. Nevertheless, the shape of many nuclei of adhering cells was more rounded in contrast to more elongated cell nuclei in native tendon. It is speculative, but this observation could suggest a less differentiated or a more synthetically-active tenoblast-like phenotype. Tendon-derived xECM has shown to present inductive stimuli, enforcing stem cells’ differentiation into a tenogenic lineage [39]. However, we found also some adipogenically-differentiating cells colonizing the implant, which possibly derive from the subcutaneous compartment of the host. The HE staining clearly revealed this ingrowth of groups of adipocytes and neoformation of univacuolar from multivacuolar adipocytes in some samples.

This observation of a more uniform cell distribution in vivo compared to in vitro might be a result of superior and more continuous nutrition of the constructs compared with the in vitro culture conditions, the inflow of host-derived growth factors and the presence of many host-derived and highly migratory cells in the subcutaneous compartment. Supporting this hypothesis, blood vessels could be shown containing smooth muscle-like SMA-positive cells, which are indicative of tunica media formation localized in the capsule region, very similar to the vessels in the epitenon of native tendon. Higher cell numbers could be detected at the ends of the scaffolds: hence, cell immigration seemed to proceed along the collagen fiber bundles starting at both ends of the scaffold, where small lanes of looser connective tissue-containing cells (including the above-mentioned adipocytes) became evident. Histological results evaluated by scoring reflected superior tissue quality in the constructs preconditioned in vitro with human tenocytes compared to cell-free implanted xECM. This might be influenced by paracrine stimulatory factors and freshly-synthesized ECM components released and deposed by the colonizing human tenocytes seeded on the xECM.

Nevertheless, despite the in vivo model used showing tendon-like tissue formation and suggested tenogenesis, one has to consider that this model is only an ectopic one with limited and undirected biomechanical stimulation of the tendon constructs, as well as with nutritive conditions in the subcutaneous compartment differing from those usually presented by the natural peritendinous tissues. 

## 4. Materials and Methods

### 4.1. Human Tenocyte Isolation and Expansion

Human primary hamstring tendon-derived tenocytes were isolated from the midsubstance of musculus (M.). semitendinosus, M. semimembranosus and M. gracilis tendons according to our previous studies [40,41]. Nine healthy middle-aged male and female donors (mean age: 36.4 years) were included. Usage of cells was approved by the Charité—Universitätsmedizin Berlin review board (30 August 2010) for experiments with human-derived tissues (EA4-033-08). After removal of the surrounding connective tissue, 2 × 3–4 mm pieces of tendons were cultured in growth medium (Ham’s F-12/DMEM 1:1 containing 10% fetal calf serum (FCS), 50 IU/mL streptomycin, 50 IU/mL penicillin, 0.5 µg/mL partricin, 1% essential amino acids, 2 mM l-glutamine (all: Biochrom AG, Berlin, Germany) and 25 µg/mL ascorbic acid (Sigma-Aldrich, Munich, Germany)) at 37 °C and 5% CO_2_. After 10–14 days, tenocytes continuously emigrated from these explants and adhered to Petri dishes. At 70% confluence, cells were removed using 0.05% trypsin/1.0 mM ethylenediaminetetraacetic acid (EDTA) (Biochrom AG) and were expanded in a monolayer culture (passage 4–6) to obtain enough cells for the cell culture seeding experiments. 

### 4.2. Porcine AS Tendon Decellularization

Porcine AS tendons were explanted post mortem from seven 3–6 month-old hybrid pigs finalized in the experimental animal unit of the Charité-University of Medicine at the end of other foreign cardiovascular projects and were immediately frozen in aqua dest. at −20 °C until use. 

Tendons were decellularized using a protocol comprising the sequential use of three different decellularization buffers: (1) 1% sodium dodecyl sulfate (SDS Sigma-Aldrich), 0.2% sodium azide (Merck, Darmstadt, Germany), 5 mM EDTA (pH 8.0, Sigma-Aldrich), freshly-added 0.4 mM phenylmethanesulfonyl fluoride (Sigma-Aldrich) and cOmplete™ Mini Protease Inhibitor Cocktail Tablet /10 mL decellularization buffer (Roche, Mannheim, Germany) for 24 h at room temperature (RT); (2) 0.05% trypsin/0.053 mM EDTA for 24 h at RT and (3) 3% Triton-X 100 (Sigma-Aldrich) for 24 h at 37 °C. The buffers were adapted from the modified protocols of Deeken et al. and Gilbert et al. [42,43]. Finally, the tendons were sterilized by incubating 30 min in 70% ethanol at RT. Then, they were rinsed three times for 5 min in pyrogen-free water and incubated subsequently for 24 h in aqua dest. before being stored at −20 °C. Samples of tendons were compared histologically before and after treatment to confirm complete cell removal. 

### 4.3. Recellularization of Tendon and Seeding of PGA

Decellularized tendons (1.0 × 0.5 × 0.3 cm) were reseeded with human hamstring tenocytes under dynamical conditions. Frozen tendons were thawed slowly until achieving RT and preconditioned by immersion in FCS for 3 h [44]. For seeding, the cell suspension (density 2 × 10^6^ cells (passage 4–6)/1 × 0.5 × 0.3 cm-long piece of AS tendon or PGA) was combined with the tendon in a bioreactor filter tube (TPP, Trasadingen, Switzerland) under orbital tilting conditions at 36 rpm in a tube roller (digital tube roller Stuart SRT9D, Bibby Scientific, Stone, Staffordshire, UK) at 37 °C and 5% CO_2_. Every 3 days, growth medium was changed, and cultures were maintained for 1–3 weeks.

### 4.4. Vitality Testing

Small pieces of recellularized tendon were incubated in 5 µL/mL fluorescein diacetate (FDA, Sigma-Aldrich, 3 mg/mL dissolved in acetone (stock solution)) and 1 µL/mL ethidium bromide (EtBr, 10 mg/mL, Carl Roth, Karlsruhe, Germany) for 10 min at 37 °C. Vital cells were green and dead cells were red, as visualized using fluorescence microscope Axioskop 40 FL (Carl Zeiss, Jena, Germany) and Cell^D imaging software (Olympus Soft Imaging Solutions GmbH, Muenster, Germany).

### 4.5. Histological Stainings

Samples were fixed in 4% paraformaldehyde (PFA). Cryo-sections (thickness: 13 µm) and deparaffinized paraffin-sections (thickness: 5 µm) were stained with HE. Sections were immersed for 4 min in Harris hematoxylin solution (Sigma-Aldrich), rinsed in water and counterstained for 4 min in eosin (Carl Roth GmbH, Karlsruhe, Germany). After rinsing with aqua dest., samples were subsequently dehydrated in an ascending alcohol series. Then, the sections were embedded with Entellan (Merck, Darmstadt, Germany) and analyzed by a microscope (Axioskop 40 FL, Carl Zeiss) and Cell^D imaging software. Photos of the sections were taken using an Olympus camera XC30 (Olympus Soft Imaging Solutions GmbH, Muenster, Germany). 

The slices were stained with 4′,6′-diamidino-2-phenylindol (DAPI, Roche, Mannheim, Germany) to analyze cell distribution and numbers. Samples were diluted in 0.1% Triton-X 100 for 45 min in a dark humid chamber and subsequently embedded in Fluoromount G (SouthernBiotech, Birmingham, AL, USA) before being assessed by fluorescence microscopy. Cell nuclei were stained using nuclear fast red aluminum sulfate solution (Carl Roth) for 5 min.

For Alcian blue (AB) staining, the sections were incubated for 3 min in 1% acetic acid (Carl Roth) and then stained 30 min in 1% AB (Carl Roth). Subsequently, they were rinsed in 3% acetic acid. Counterstaining of cell nuclei was performed using nuclear fast red aluminum sulfate solution (Carl Roth) for 5 min.

### 4.6. Nude Mice Xenograft Model

Human tenocytes were cultured for 7 days dynamically on 1 × 0.5 cm-diameter PGA meshes (PGA biofelt, 65 mg/cc, Concordia Medical, Warwick, RL, USA) or decellularized tendon xECM, as described above. Constructs were implanted subcutaneously into the dorsal subcutaneous space of female 4-8-week-old athymic (nude) mice (Charles River, Sulzfeld, Germany). The mice received Rimadyl (4 mg/kg body weight, Pfizer, Karlsruhe, Germany) subcutaneously as analgesic premedication 20 min before being anesthetized using isoflurane (Forene 100%, Abbott, Wiesbaden, Germany) and after 24 h once again as analgesia post-surgery. The back skin was incised (1 cm) under sterile conditions; a pocket beneath the incision was opened, and the construct was inserted. The incision was closed with 6-0 Prolene (Ethicon, Bridgewater, NJ, USA). The animals were divided randomly into the different groups, depending on whether the scaffolds (xECM or PGA) were seeded or not, before being implanted for six or 12 weeks (*n* = 4–9 in each group and at each time point). The cell-free scaffolds were treated in a similar manner. Six or 12 weeks post implantation of the constructs, animals were sacrificed. The explanted constructs were macroscopically scored using a self-designed scoring system (entailing the color, the constitution of the constructs, structural characteristics and maintenance of original size adapted from [38] (Table 1) and then measured and documented photographically using a Canon EOS500D camera with a Canon EF-S60 mm macro objective (Canon, Krefeld, Germany). 

Subsequently, they were fixed for 15 min in 4% paraformaldehyde (Santa Cruz Biotechnology Inc., Dallas, TX, USA), dehydrated overnight in 30% sucrose solution before being embedded in Tissue-Tek^®^ O.C.T (Sakura Finetek USA, Inc., Torrance, CA, USA) or embedded in paraffin. Cryo-sections were prepared, histologically stained and analyzed using a histological scoring system (Table 2).

### 4.7. Total DNA and GAG Content Measurement

The DNA contents of native, decellularized and dynamically-recellularized tendons were measured via the CyQuant assay (Invitrogen, Carlsbad, CA, USA) according to the user manual by using calf thymus DNA as a standard. The samples were digested with a proteinase K solution (10 mg/mL dissolved in 50 mM Tris/HCl, 1 mM EDTA, 0.5% Tween20, pH 8.5) for 16 h at 55 °C and centrifuged for 30 min at 10,000× *g*. The supernatants were stored by 4 °C. Based on this assumption that each cell contains around 7.7 pg DNA [45], cell content in the tendons was estimated. To quantify the GAG contents, the probes were diluted in phosphate-buffered EDTA (PBE) (100 mM Na_2_HPO_4_ and 5 mM EDTA, pH 8.0). Then, the dimethyl methylene blue (DMMB, AppliChem, Darmstadt, Germany) dyeing solution (8.9 mM DMMB hydrochloride in 600 mg glycerin, 467 mg NaCl and 200 mL aqua dest.) was added. The absorption shift from λ = 525 to 595 nm was measured immediately. GAG contents were calculated by using chondroitin sulfate as a standard.

### 4.8. Immunolabeling of α-Smooth Muscle Actin and Anti-Human Nuclei in the Reseeded Samples

To assess the formation of blood vessels in the constructs implanted in the nude mice immunolabeling of α-smooth muscle actin *(*αSMA) was performed, which is a marker of smooth muscle cells and also present in pericytes and myofibroblasts. Anti-human nuclei immunolabeling was performed to differentiate human cells from murine cells in the constructs explanted from the murine host.

Specificity of the anti-nuclei staining was checked using murine fibroblasts and human tenocytes in monolayer (co-)cultures. Hamstring-derived human tenocytes were characterized by anti-scleraxis and -tenascin C immunolabeling (see the graphical abstract). Deparaffinized sections (5 µm thick) or cells seeded on poly-l-lysin-coated glass slides fixed in 4% PFA were washed with Tris-buffered saline (TBS: 0.05 M Tris, 0.015 M NaCl, pH 7.6), before being incubated with protease-free donkey serum (5% diluted in TBS with 0.1% Triton-X 100 for cell permeabilization) for 20 min at RT. Subsequently, samples were incubated with primary antibodies (αSMA: mouse-anti-human 1:50, Sigma-Aldrich, Munich, Germany), mouse-anti-human nuclei (1:30, Millipore, Darmstadt, Germany), rabbit-anti-human scleraxis (1:30, Acris Laboratories, Hiddenhausen, Germany) or mouse-anti-human tenascin C (1:50, GeneTex Inc., Biozol, Eching, Germany) for 1 h at RT in a humid chamber. Samples were rinsed with TBS before incubation with Cy3-coupled donkey-anti-mouse or Alexa488-coupled donkey-anti-rabbit secondary antibodies (Invitrogen, Carlsbad, CA, USA, diluted 1:200 in Tris buffered saline (TBS)), respectively, for 1 h at RT. Cell nuclei were counterstained using DAPI. Labeled sections were washed three times with TBS, before mounting with Fluoromount G mounting medium (Southern Biotech, Biozol Diagnostica, Eching, Germany) and examined using a TCS SPE II confocal laser scanning microscope (Leica, Leica Microsystems, Wetzlar, Germany).

### 4.9. Statistical Analysis

All values were expressed as the mean with the standard deviation using GraphPad Prism 5, (GraphPad software Inc., San Diego, CA, USA). Data based on score values (ordinal scaled data, e.g., scoring values) were analyzed using the Kruskal–Wallis test followed by Dunn’s post-hoc test. In addition, the Mann–Whitney test was used to compare two separate groups.

Parametric data were analyzed using the unpaired two-tailed *t*-test (e.g., cell content), one-way ANOVA with Holm–Sidak adjustment. The Kruskal–Wallis test was used followed by Dunn’s post-hoc test for non-parametric data (e.g., DNA and GAG contents). Statistical significance was set at a *p-*value ≤ 0.05.

## 5. Conclusions

In summary, the xECM exerted a high biocompatibility, superior to that of PGA, which is a well-established synthetic polymer used in the clinic for other approaches [46,47,48]. Generally, constructs seeded with tenocytes showed a superior histological tissue quality and a slightly higher cell content compared with unseeded constructs. Moreover, originally-seeded human tenocytes could be identified in the explants. This observation underlines the advantage of preconditioning a xECM with suitable cells. However, the question about whether these human tenocytes growing on the porcine xECM might change their phenotype should be addressed in future experiments, e.g., by testing the expression of typical tendon markers such as scleraxis. Possibly, the use of autologous cells might present additional advantages; however, this approach could not be tested in the mice model here. Therefore, future in vivo experiments should be performed in an orthotopic model with autologous tenocytes to investigate the functionality of reseeded xECM scaffolds, and the biomechanical strength should be tested to prove functionality.

## Figures and Tables

**Table 1:** Macroscopical scoring system for tendon explants.

Macroscopical Scoring	Points
**color**	
white, glossy	2
rose, rough	1
other color	0
**surface structure**	
smooth, tight, intact	2
rough	1
surface with clefts and holes	0
**shape**	
like before implantation	2
unregular, tight	1
diffuse, smooth	0
**size**	
similar like before implantation	2
shrunken or swollen	1
nearly disappeared	0
maximum value	**8**

Adapted to tendon from Lohan et al. [38].

**Table 2:** Histological scoring (HE, AB) system for tendon explants.

Histological Scoring	
**ECM**	**Points**
dense, parallel organized fiber bundles	2
partly dense, partly loose and unorganized	1
loose unorganized cell-ECM composition	0
**Proteoglycans (Alcian Blue Staining)**	
normal	2
focally elevated or impaired	1
generally elevated or impaired	0
**Cells**	
**Cellularity**	
normal (5–10% of tissue volume)	2
slight or focal hypo-/hyper-cellularity	1
severe hypo-/hyper-cellularity	0
**Distribution**	
homogeneous	1
focal areas with higher/lower cell density	0
**Cell Orientation**	
in rows between ECM bundles	2
focally not orientated (10–50%)	1
>50% not orientated	0
**Morphology of Cell Nuclei**	
mostly elongated	2
mixed: 10–30% round/oval shaped	1
mostly round/oval-shaped, euchromatically or polymorph heterochromatically, tenoblasts or other extrinsic fibroblasts	0
**Degeneration/Metaplasia (Alcian Blue)**	
none	1
edema, fatty tissue, cartilage, bone or fibrinoid inclusion, cell debris deposition, fiber destruction	0
**Vascularization (Within the Explant)**	
barely detectable, very low (physiological)	1
locally or generally increased	0
**Infiltrating Inflammatory Cells (Neutrophils, Macrophages, Lymphocytes, Foreign Body Cells)**	
no	2
moderate/locally/focally	1
severe, generalized	0
maximum value	15

Adapted to tendon from Lohan et al. [38].

## Supplementary Materials

Supplementary materials can be found at http://www.mdpi.com/1422-0067/19/7/2059/s1.

## Abbreviations

αSMA	α-smooth muscle actin
AB	Alcian blue
AS	Achilles
DAPI	4′,6′-diamidino-2-phenylindol
DMMB	dimethyl methylene blue
ECM	extracellular matrix
EDTA	ethylenediaminetetraacetic acid
Etbr	ethidium bromide
FCS	fetal calf serum
FDA	fluorescein diacetate
GAG	glycosaminoglycan
HE	hematoxylin/eosin
M.	musculus
MSC	mesenchymal stromal cells
PBE	phosphate-buffered EDTA
PFA	paraformaldehyde
PGA	polyglycolic acid
PI	propidium iodide
RT	room temperature
SDS	sodium dodecyl sulfate
TBS	Tris-buffered saline
sGAG	sulfated glycosaminoglycans
