# Supplementary material for: Tenogenesis of Decellularized Porcine Achilles Tendon Matrix Reseeded with Human Tenocytes in the Nude Mice Xenograft Model
Source: Int J Mol Sci. 2018 Jul 15;19(7):2059. doi: 10.3390/ijms19072059 (PMC6073795; doi:10.3390/ijms19072059)
Supplement: Supplementary file 1 [file ijms-19-02059-s001.pdf]

Supplemental Material

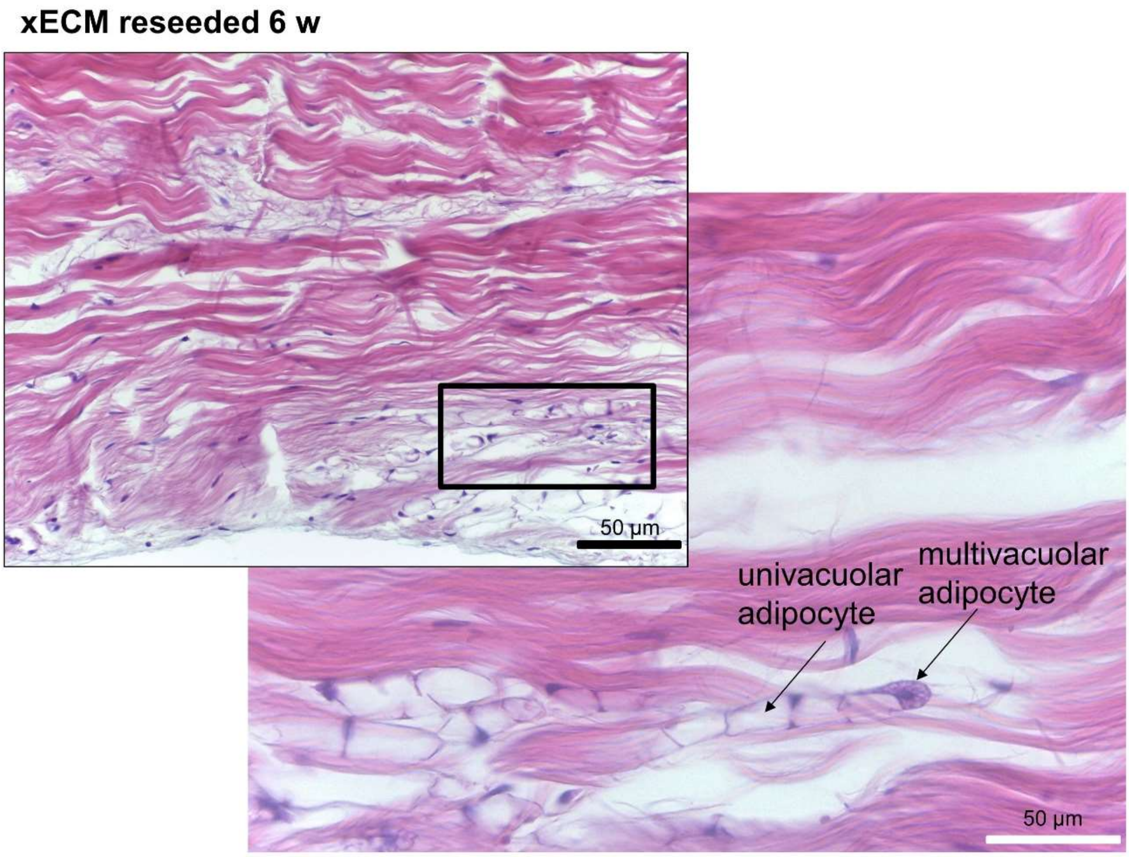

Supplemental Figure S1. Developing adipocytes in decellularized xECM seeded with human tenocytes after 6 weeks *in vivo*. Rows of unipolar adipocytes arise between ECM fiber bundles with a multipolar adipocyte ad its end after 6 weeks (w).
